# Large-scale analysis of *CDH1* mutations defines a distinctive molecular subset with treatment implications in gastric cancer

**DOI:** 10.1038/s41698-024-00694-8

**Published:** 2024-09-30

**Authors:** Jingyuan Wang, Joanne Xiu, Francesca Battaglin, Hiroyuki Arai, Shivani Soni, Wu Zhang, Richard M. Goldberg, Philip A. Philip, Andreas Seeber, Jimmy J. Hwang, Anthony F. Shields, John L. Marshall, Igor Astaturov, Tianshu Liu, A. Craig Lockhart, W. Michael Korn, Lin Shen, Heinz-Josef Lenz

**Affiliations:** 1 Department of Medical Oncology, Cancer Center, Zhongshan Hospital, Fudan University, Shanghai, China; 2 Division of Medical Oncology, Norris Comprehensive Cancer Center, Keck School of Medicine, University of Southern California, Los Angeles, CA USA; 3 Key Laboratory of Carcinogenesis and Translational Research (Ministry of Education/Beijing), Department of Gastrointestinal Oncology, Peking University Cancer Hospital and Institute, 52 Fucheng Road, Haidian District, Beijing, 100142 China; 4 Caris Life Sciences, Phoenix, AZ USA; 5 West Virginia University Cancer Institute, Morgantown, WV USA; 6 Department of Oncology, Karmanos Cancer Institute, Wayne State University, Detroit, MI USA; 7 Department of Hematology and Oncology, Comprehensive Cancer Center Innsbruck, Medical University of Innsbruck, Innsbruck, Austria; 8 Levine Cancer Institute, Carolinas HealthCare System, Charlotte, NC USA; 9 Ruesch Center for The Cure of Gastrointestinal Cancers, Lombardi Comprehensive Cancer Center, Georgetown University Medical Center, Washington, DC USA; 10 Fox Chase Cancer Center, Philadelphia, PA USA; 11 University of Miami/Sylvester Comprehensive Cancer Center, Miami, FL USA

**Keywords:** Gastric cancer, Targeted therapies

## Abstract

Although histological and molecular classifications have been extensively studied for gastric cancer (GC), targeted therapies for GC remain limited. *CDH1* mutations (MT) are characteristic of genomically stable GC and are associated with poor prognosis, but lack effective or targeted therapies. Here, we showed the overall mutation frequency of *CDH1* was 9.7% (155 of 1596). *CDH1*-MT GC showed significantly lower rates of PD-L1 positivity (CPS score ≥1) than *CDH1*-wildtype (WT) GC (56.7% vs. 73.3%, *p* < *0.05*). Compared to *CDH1*-WT GC, mutations of *ARID1A*, *WRN*, *POT1*, *CDK12*, and *FANCC* were significantly higher, while *TP53* and *APC* were significantly lower in *CDH1*-MT GC (*p* < 0.05); The rates of *KRAS* and *HER2* amplifications were significantly lower, while *CRKL* and *IGF1R* amplifications were significantly higher in *CDH1*-MT GC, compared to *CDH1*-WT GC (*p* < 0.05). Frequently altered genes in *CDH1*-MT GC were especially enriched in DNA damage repair and cell cycle checkpoint pathways. Inhibition of E-cadherin sensitized GC cell lines to PARP and Wee1 inhibitors by disrupting DNA damage repair pathway and cell cycle checkpoint. This is the largest study to investigate the distinct genomic landscape of *CDH1*-MT GC. Our data indicated GC patients with *CDH1* mutations could potentially benefit from agents targeting PARP and Wee1.

## Introduction

Gastric cancer (GC) is a highly heterogeneous disease with marked histological and molecular diversity, which has been a major obstacle in identifying effective novel drug treatments. Except for surgery, chemotherapy remains the major option for treating most patients with advanced GC. Currently, drugs targeting human epidermal growth factor receptor 2 (HER2), vascular endothelial growth factor 2 (VEGFR2), CLDN18.2, and immunotherapy have been found to significantly prolong the overall survival, but only appear to be effective in sub-populations while the majority of cases continue to manifest drug resistance to targeted therapies^1–4^. Thus, there is a significant clinical need to discover novel therapeutic targets for GC.

Over the past decades, the worldwide effort to characterize the molecular features of GC has led to the identification of multi-driver genes/pathways, including cell adhesion and chromatin remodeling genes^5–9^. According to The Cancer Genome Atlas (TCGA) project, four subgroups of GC were defined: Epstein-Barr virus (EBV) positive, microsatellite instability (MSI), genomically stable (GS), and chromosomal instability (CIN)^7^. These findings both significantly expanded our understanding of the tumorigenesis and development of GC and provided a framework for dissecting the population with GC and the opportunity for exploiting the value of targeted therapies that have mechanisms of action that will likely be effective in subsets of patients with GC. However, such genetic classifications have not been translated into effective targeted approaches in clinical practice.

E-cadherin, which is encoded by *CDH1*, is a well-known calcium-dependent cell adhesion molecule. It is a tumor suppressor and plays a major role in maintaining epithelial architecture and cell polarity^10^. E-cadherin loss in various cancers promotes the metastasis of cancer cells by the dysregulation of intercellular adhesion between epithelial cells^11^. *CDH1* mutation is one of the mechanisms of E-cadherin loss, which is sufficient to induce tumorigenesis in preclinical models^12^. Earlier work on *CDH1* mutations in GC mainly focused on germline mutations in GC patients with family history, which is a known risk factor for hereditary diffuse GC (HDGC)^13^. In the sporadic context, *CDH1* somatic mutations were highly prevalent in diffuse GC (DGC) and the TCGA GS subgroup and could predict worse prognosis independently^14^. However, effective treatment for this subgroup is currently limited. Therefore, it is imperative to explore the molecular features of *CDH1*-mutated (MT) GC and identify potential druggable targets for this cohort.

This study was designed to compare the molecular features and enriched pathways between *CDH1*-MT and wildtype (WT) GC based on a large genomic database generated from 1596 samples. We also identified the potential druggable targets/pathways in *CDH1*-MT GC and then explored their potential by in vitro experiment.

## Results

### Overview of the genetic landscape of *CDH1* mutations of GC in the CARIS databank

**Fig. 1:**
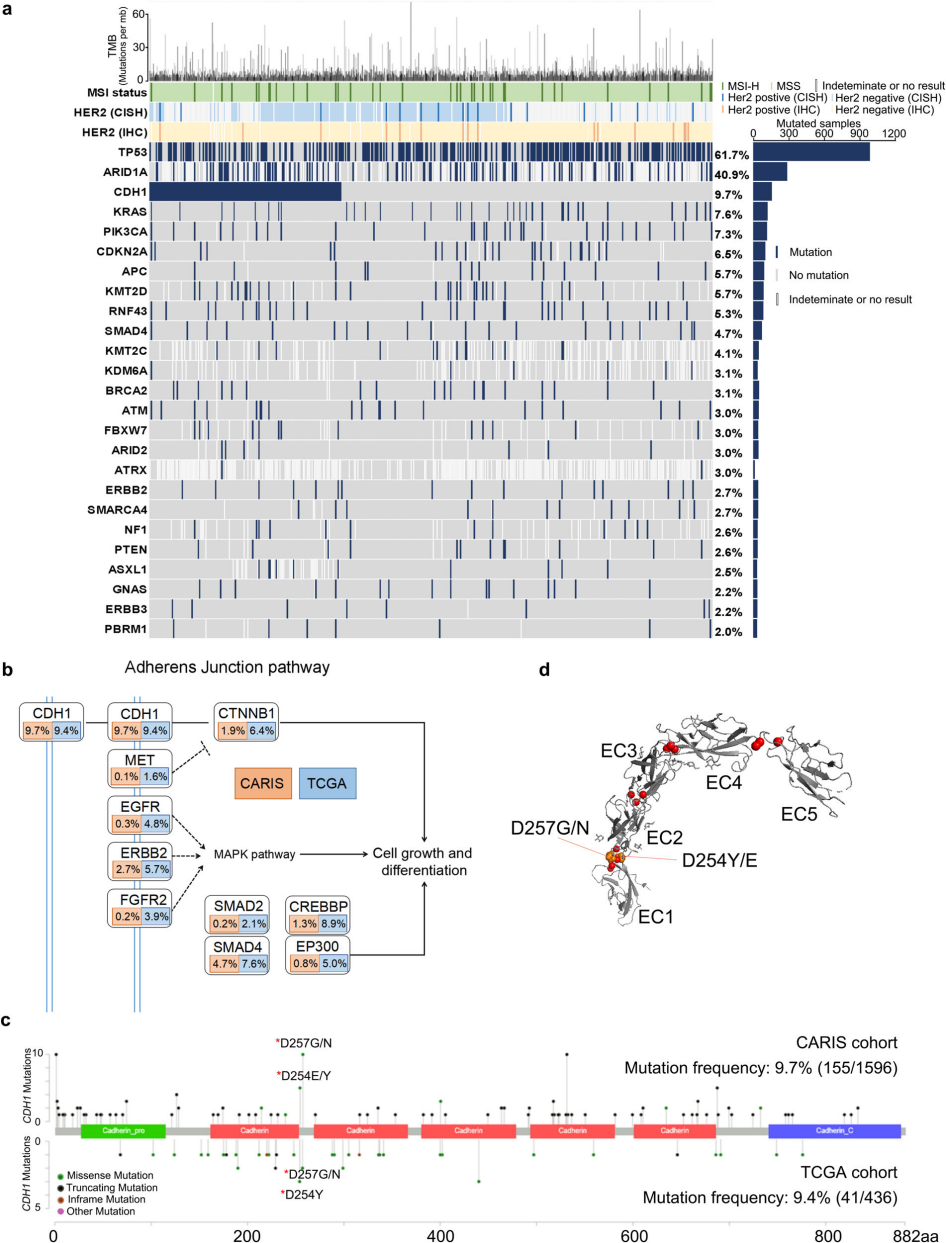
Summary of the somatic mutations and Indels identified in all 1596 patients. **a** Mutational landscape of the top 25 frequently mutated genes with different clinicopathological features of GC patients from the CARIS database. Blue, genetic alteration (nonsynonymous SNV, stop-gain, indel); Gray, no mutation; White, indeterminate or no result. **b** The schematic of major genetic alterations in cell adhesion pathway, the frequencies (%) in the CARIS cohort (orange) and TCGA cohort (blue) were shown. **c** Distribution of *CDH1* mutations according to *CDH1* domains. The red stars indicated hotspot mutations identified in both TCGA and CARIS cohorts. **d** Structure of E-cadherin. Three-dimensional structure visually showed these hotspot mutations (Orange, D247G/N, D254E/Y) could cause significant functional perturbations of critical calcium-binding sites (Red) in the EC1-2 junction.

To gain insight into the genetic alterations of GC, we performed targeted sequencing in a cohort of 1596 patients. Clinical and pathological features were summarized in Supplementary Table 1. Overall, 3444 somatic mutations and 1438 copy number amplifications were detected. As demonstrated in Fig. 1a, the most frequently mutated genes were *TP53* (61.7%), followed by *ARID1A* (40.9%), *CDH1* (9.7%), *KRAS* (7.6%) and *PI3KCA* (7.3%).

Abnormal cell adhesion has been identified as one of the most significantly enriched biological processes among the mutated genes in GC^6^, for which *CDH1* is considered to be a well-known mediator. We further compared the mutation rates of genes involved in cell adhesion between the CARIS and TCGA cohorts (Fig. 1b). Most genes showed significantly lower mutation rates in the CARIS cases as compared to those in TCGA cases. For *CDH1*, the mutation rates were consistent between CARIS and TCGA cohorts (9.7% vs. 9.4%), suggesting that *CDH1* may play an important role in driving tumorigenesis in a subset of patients with GC.

*CDH1* encodes a calcium-dependent cell adhesion glycoprotein (E-cad, E-cadherin) and has five extracellular cadherin domains (EC1-EC5)^15^. Mutations in the EC1-2 and EC2-3 junctions are known to cause improper cadherin localization and diminished cell adhesion^16^. For this reason, we next analyzed where the hotspot mutations in *CDH1* are located. A total of 178 *CDH1* mutations (111 different *CDH1* mutations) were identified among 155 patients, including 47 frameshift mutations, 26 missense mutations, 26 nonsense mutations, and 79 splicing mutations. All of these *CDH1* mutations were predicted to be pathogenic or likely pathogenic. Similar to the observations in the TCGA database, most of the *CDH1* mutation sites were located in the EC1 domain (Fig. 1c). *CDH1* hotspot mutations (D257G/N, D254E/Y), shown in the TCGA cohort, which were located in the EC1-2 junctions, were also detected in 15 patients from the CARIS cohort. As shown in Fig. 1d, these two hotspot mutations could disrupt the cadherin–cadherin interactions directly.

### Correlation of *CDH1* mutations to the clinicopathological features

To better understand the features of the *CDH1*-MT cohort, we compared the clinicopathological parameters between *CDH1*-MT and WT tumors. As shown in Table 1, *CDH1* mutations were significantly associated with non-gastroesophageal junction (GEJ) cancer (12.4% vs. 1.5%), younger age (median 57 vs. 64 years old), female (12.7% vs. 8.0%) and diffuse histology (26.8% vs. 1.4%) GC (*p* < 0.05). Meanwhile, both younger (median 60 vs. 68 years old) and diffuse histology (21.2% vs. 4.7%) GC patients showed more frequent *CDH1* mutations in the TCGA cohort as well (*p* < 0.05).

**Table 1:** Correlation of *CDH1* mutation to clinicopathological features of GC

Clinicopathologic feature		CARIS (*N* = 1596)			TCGA (*N* = 436)		
		*CDH1*-MT (%)	*CDH1*-WT (%)	*p*	*CDH1*-MT (%)	*CDH1*-WT (%)	*p*
Age^a^	Median	57	64	<0.001^b^	60	68	0.01^b^
		(15–90)	(12–94)		(41–79)	(30–90)	
	Female	55	64	0.002^b^	58.5	69	0.073
		(27–90)	(12–94)		(41–78)	(34–90)	
	Male	59.5	64	0.051	60	67	0.065
		(15–86)	(12–94)		(43–79)	(30–90)	
Gender	Female	73 (12.7)	502 (87.3)	0.0035^b^	14 (9.0)	144 (91.0)	0.728
	Male	82 (8.0)	939 (92.0)		28 (10.0)	251 (90.0)	
Type	Non-GEJ	149 (12.4)	1053 (87.6)	<0.001^b^	-	-	-
	GEJ^c^	6 (1.5)	388 (98.5)		-	-	
Lauren subtype^d^	Diffuse	33 (26.8)	90 (73.2)	<0.001^b^	18 (21.2)	67 (78.8)	<0.001^b^
	Intestinal	1 (1.4)	72 (98.6)		9 (4.7)	181 (95.3)	
	Mixed or unclear	121 (8.6)	1279 (91.4)		15 (9.3)	146 (90.7)	

^a^Median (range).

^b^A two-sided *p* < 0.05 was considered statistically significant. *p* was calculated by chi-square test, unpaired two-tailed t-test, nonparametric test, or one-way analysis of variance separately.

^c^*GEJ* gastroesophageal junction.

^d^TCGA cohort: Diffuse subtype includes signet ring cell carcinoma; Intestinal subtype includes tubular stomach adenocarcinoma, papillary stomach adenocarcinoma, and mucinous stomach adenocarcinoma.

**Fig. 2:**
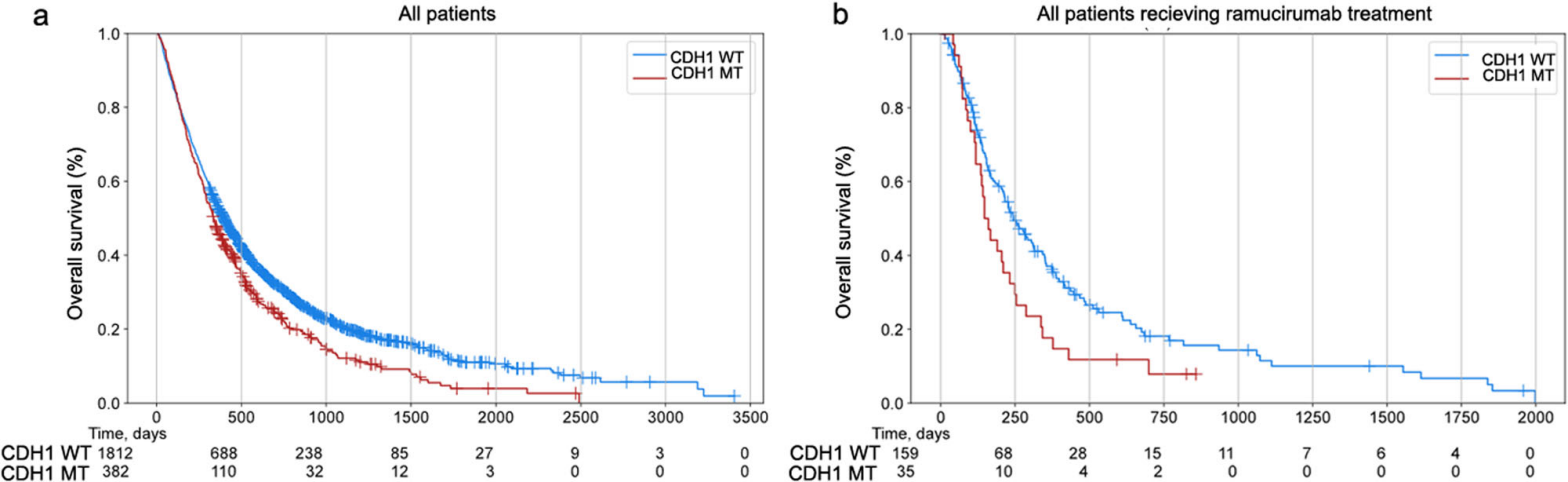
The impact of CDH1 mutations on clinical outcomes in GC patients. **a** The prognosis value of CDH1mutations in GC. **b** The association of CDH1 mutations with ramucirumab-related survival in GC.

Overall, *CDH1* mutations were associated with poor prognosis [median overall survival (OS): 11.23 vs. 13.27 months, HR = 1.24, 95% CI: 1.10–1.41, *p* < 0.001, Fig. 2a]. The association of *CDH1* mutations with poor prognosis remained significant in microsatellite stable (MSS) GC patients (10.9 vs. 12.97 months, HR = 1.23, 95% CI 1.08–1.41, *p* = 0.002, Supplementary Fig. 1a). In ramucirumab-treated GC patients, patients with *CDH1* mutations (*n* = 159) exhibited significantly shorter OS (median: 4.90 vs 8.13 months, HR = 1.54, 95% CI: 1.03–2.27, *p* = 0.034, Fig. 2b), compared to patients with *CDH1*- WT tumors (*n* = 35).

### Molecular alterations of *CDH1*-MT GC vs. *CDH1*-WT GC

**Fig. 3:**
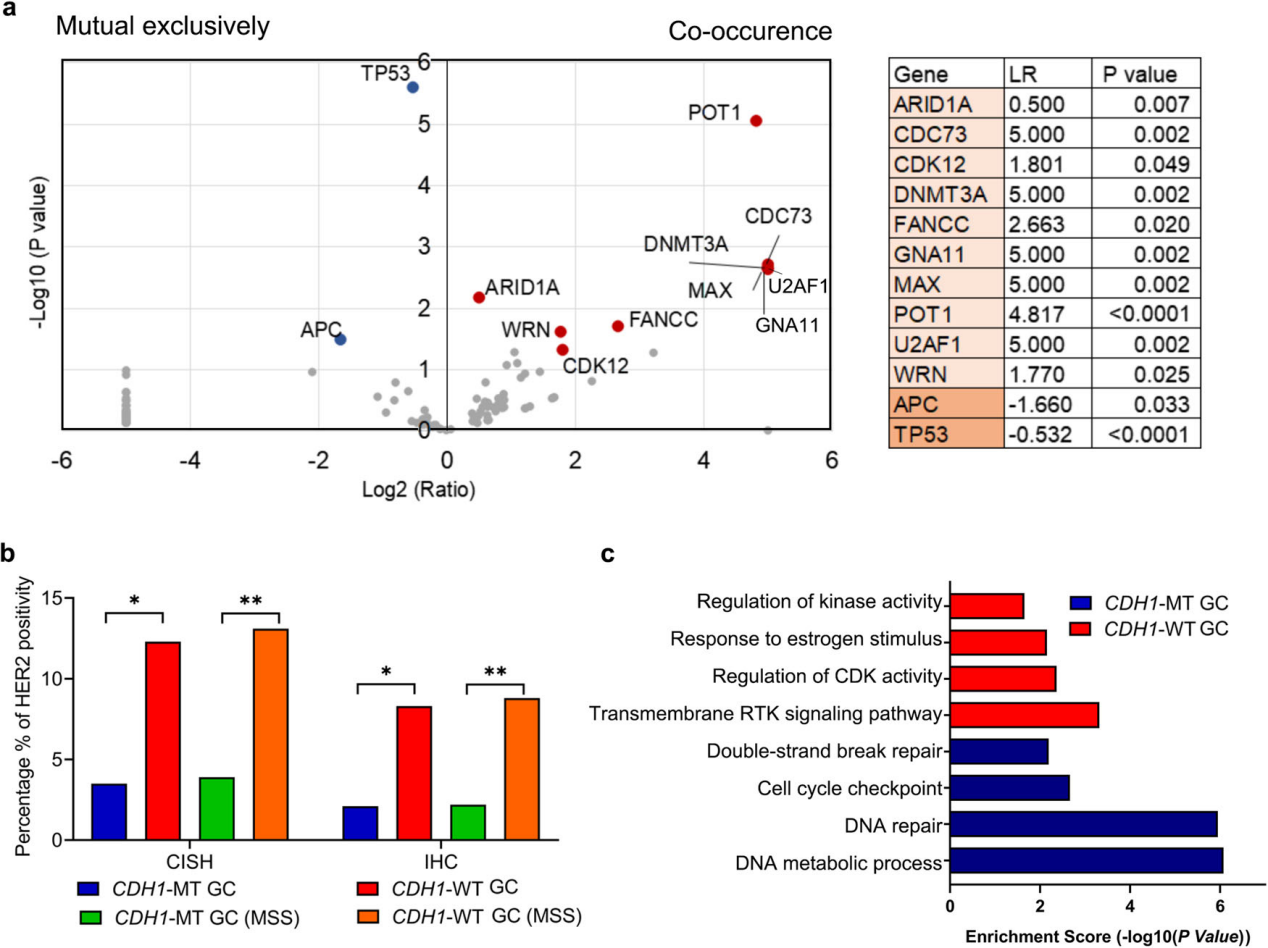
Distinct molecular subtype identified by *CDH1* mutations in GC. **a** Mutual exclusivity and co-occurrence between *CDH1* mutations and cancer-related gene mutations. *x*-axis indicates co-occurring mutations (right) and mutually exclusive mutations (left), *y*-axis indicates the significance. Log2 (ratio) is a Log2-based ratio of mutation frequency in patients with *CDH1* MT/mutation frequency in patients with *CDH1* WT. The details of mutually exclusive and co-current genes with respect to *CDH1* mutations were shown left. (**b**). HER2 amplification (CISH) and expression were compared between *CDH1*-MT and WT cohorts. *p < 0.05; **p < 0.01 by one-way ANOVA or chi-squared test. **c** Several functional terms, including double-strand break repair, cell cycle checkpoint, DNA metabolic process, and DNA repair pathways, were exclusively found to be enriched in the *CDH1*-MT cohort by GO analysis, whereas regulation of CDK activity, regulation of kinase activity, transmembrane RTK signaling pathway and response to estrogen stimulus pathways were enriched in the *CDH1*-WT cohort in GC. CDK cyclin-dependent kinase, RTK receptor tyrosine kinase, GO Gene Oncology.

Co-occurrence and mutual exclusivity analysis revealed that ten genes were found to potentially co-occur with *CDH1* mutations, while only *APC* (1.9% vs. 6.2%) and *TP53* (44% vs. 63.6%) mutations tended to be mutually exclusive with *CDH1* mutations (*p* < 0.05) (Fig. 3a). The top five most frequently mutated genes were *ARID1A* 55.3% vs. 39.1%, *WRN* 2.9% vs. 0.8%, *POT1* 2.1% vs. 0.1%, *CDK12* 1.9% vs. 0.6%, and *FANCC* 1.4% vs. 0.2%. Among co-occurring mutated genes, *ARID1A* and *DNMT3A* were related to chromatin remodeling, and *CDK12*, *FANCC*, *POT1*, and *WRN* were related to DNA damage repair. Additionally, *KRAS* (2.11% vs. 7.65%) and *ERBB2* (1.39% vs. 8.60%) showed lower frequent amplification rates, while *IGF1R* (1.40% vs. 0.15%) and *CRKL* (2.1% vs. 0.36%) showed more frequent amplification rates in the *CDH1-*MT cohort, compared to the *CDH1*-WT cohort (*p* < 0.05) (Table 2). The rates of HER2 positivity detected by Immunohistochemistry staining (IHC) (2.1% vs. 8.3%) and Chromogenic in situ hybridization (CISH) (3.5% vs. 12.3%) were also lower in *CDH1*-MT cohort than those in *CDH1*-WT cohort (*p* < 0.05) (Fig. 3b), consistent with the copy number analysis. No significant difference in ARHGAP26 fusion was observed between *CDH1*-MT and WT GC (7.69% vs. 8.37%, *p* = 0.906) (Supplementary Table 2).

**Table 2:** The significant differences of amplified genes between *CDH1*-WT and MT cohort

Chr	Gene	Location	*CDH1*-MT	*CDH1*-WT	*p*
12	*KRAS*	12p12.1	2.11% (3/142)	7.65% (96/1255)	0.0204^a^
15	*IGF1R*	15q26.3	1.40% (2/143)	0.15% (2/1348)	0.0064^a^
17	*ERBB2*	17q12	1.39% (2/144)	8.60% (109/1267)	0.0038^a^
22	*CRKL*	22q11.21	2.10% (3/143)	0.36% (5/1371)	0.0072^a^

^a^A two-sided *p* < 0.05 was considered statistically significant. *p* was calculated by the chi-square test.

Next, we selected the most frequently mutated genes (mutation rate ≥ 1%) in the *CDH1-*WT and MT cohort for Gene ontology (GO) analysis. Genes related to DNA damage repair, DNA double-strand break repair, DNA metabolic processes, and cell cycle checkpoints were specifically enriched in the *CDH1-*MT cohort. Genes related to the regulation of kinase/cyclin-dependent kinase activity, response to estrogen stimulus, and transmembrane receptor protein tyrosine kinase (RTK) signaling pathway were enriched in the *CDH1-*WT cohort (Fig. 3c). The details of GO enrichment are shown in Supplementary Table 3.

**Fig. 4:**
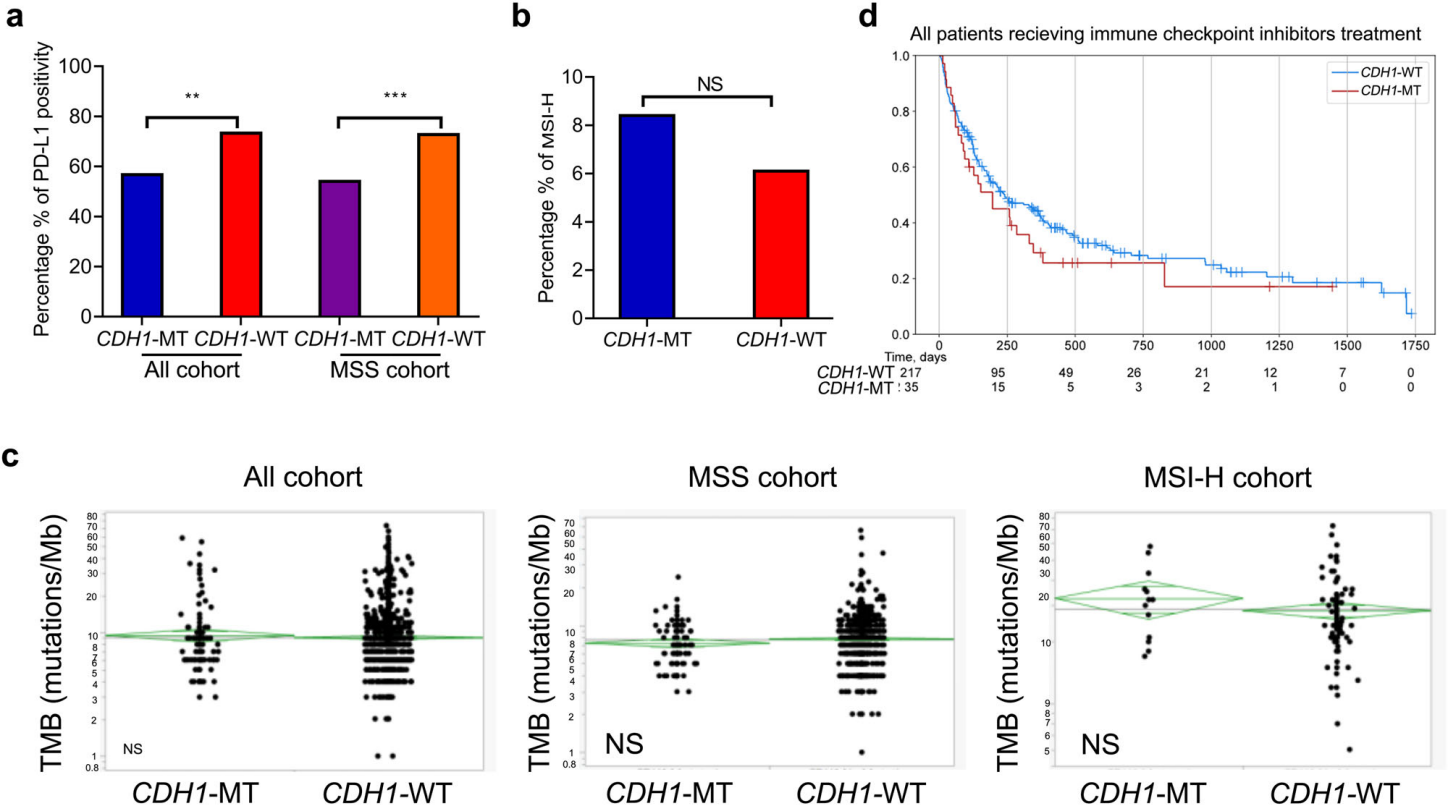
Comparison of immune profiles between *CDH1*-MT and WT GC in all cohort and MSS/MSI-H cohorts, respectively. **a** The positivity rates of PD-L1 expression were lower in the *CDH1*-MT cohort, compared to *the CDH1*-WT cohort (CPS ≥ 1). **b**, **c** No significant difference in MSI-H and TMB status was shown between *the CDH1*-WT/MT cohort. **d** The association of *CDH1* mutations with immune checkpoint inhibitor-related survival in GC patients. **p < 0.01. ***p *<* *0.001*. NS not significant.

Subsequently, we investigated the relationship between *CDH1* mutations and biomarkers predictive of response to checkpoint inhibitor treatments. As shown in Fig. 4a, *CDH1*-MT GC showed significantly lower rates of PD-L1 positivity (CPS ≥ 1) (all cohorts: 56.7% vs. 73.3%, *p* < 0.01; MSS: 54.0% vs. 72.7%, *p* < 0.001). No significant difference in the prevalence of MSI-H status was observed between *CDH1*-MT and WT GC (8.4% vs. 6.1%, *p* = 0.248) (Fig. 4b). Moreover, TMB scores also showed a similar distribution in the *CDH1*-MT GC, compared to the *CDH1*-WT GC (all cohort: median TMB 7 vs. 8 mt/MB, *p* = 0.53). Similar results were seen between *CDH1*-MT and WT GC with or without MSI-H (MSI-H: median TMB is 32 vs. 26 mt/MB, *p* = 0.26*;* MSS: median TMB 7 vs. 7 mt/MB, *p* = 0.09) (Fig. 4c). Patients with *CDH1* mutations showed numerically shorter immune-related OS (irOS), compared to those with *CDH1*-WT (all cohort: median OS 6.53 vs 8.03 months, HR = 1.24, 95%CI: 0.81–1.88, *p* = 0.319; MSS cohort: 4.77 vs 6.3 months, HR = 1.23, 95%CI: 0.78–1.93, *p* = 0.376), although this difference did not reach statistical significance (Fig. 4d, Supplementary Fig. 1b).

We further evaluated the transcriptomic features of *CDH1-*MT GC using RNA-sequencing data derived from the TCGA cohort. Interestingly, Gene set enrichment analysis (GSEA) analysis revealed that except for known EMT upregulation and apical junction, inflammatory-related pathways (myogenesis, allograft rejection, UV response downregulation, complement, KRAS signaling upregulation, IL2-STAT5) were enriched in the *CDH1*-MT GC cohort, compared to the *CDH1*-WT cohort (Supplementary Fig. 2a). Meanwhile, genes associated with the G2/M checkpoint and DNA repair pathways tended to be enriched in the *CDH1*-WT cohort (Supplementary Fig. 2b). GO and Kyoto Encyclopedia of Genes and Genomes (KEGG) analyses suggested that angiogenesis-related pathways (vasculature development, regulation of angiogenesis and chemokine signaling pathway) were significantly enriched in the *CDH1*-MT cohort (Supplementary Fig. 2c). Furthermore, we also found the infiltration of activated dendritic cells were lower in the *CDH1*-MT GC cohort, compared to the *CDH1*-WT GC cohort (Supplementary Fig. 2d).

### Downregulation of E-cadherin sensitizes GC cell lines to olaparib and AZD1775 via disruption of DNA damage repair pathways and cell cycle checkpoints

**Fig. 5:**
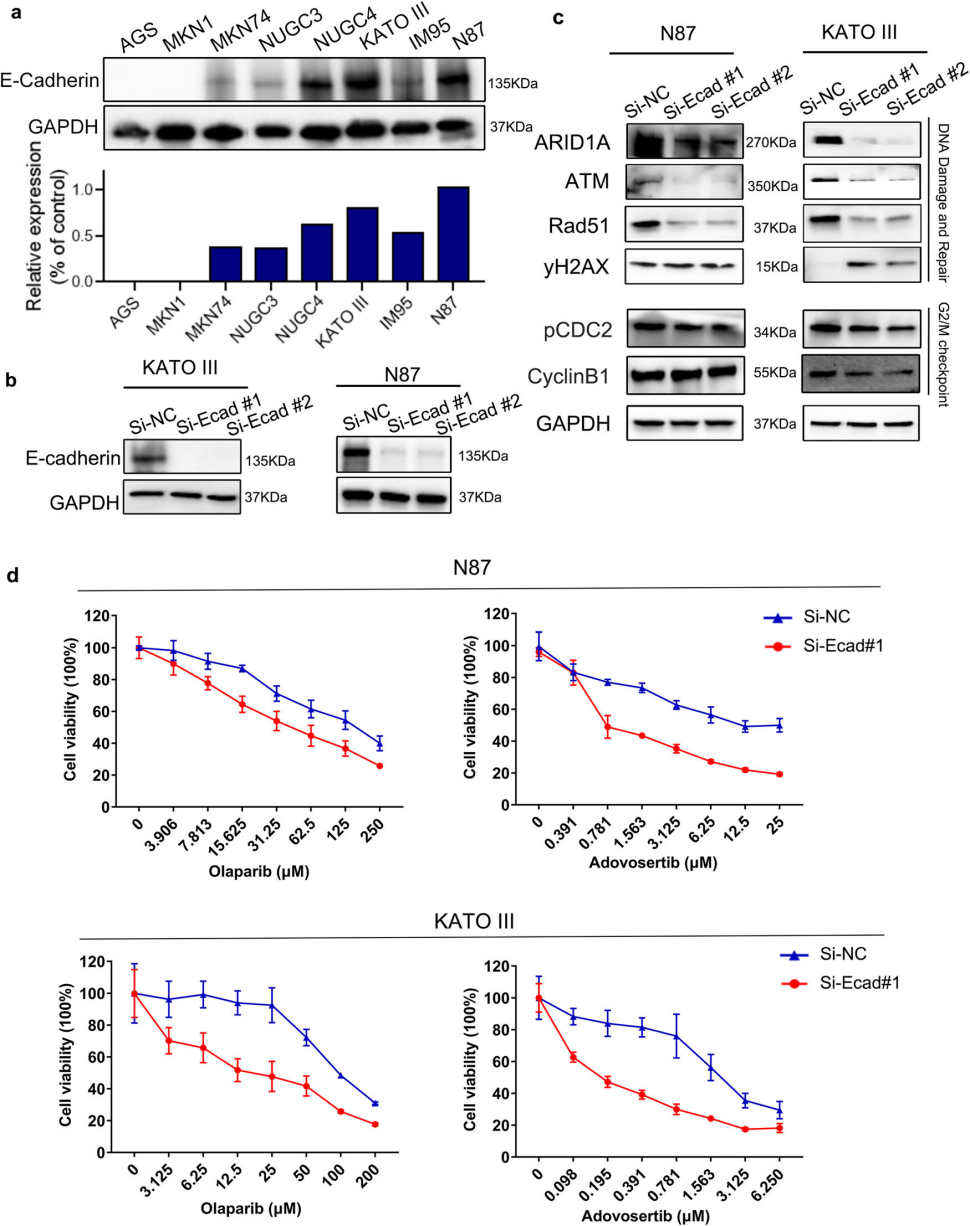
Downregulation of E-cadherin sensitized gastric cancer cell lines to PARP inhibitor and Wee1 inhibitor. **a** E-cadherin expression in eight GC cell lines was detected by western blotting (top). Images below show the protein expression levels of E-cadherin were quantified by the software Image J (below). **b** Cells transfected with Si-E-cadherin (Ecad)/Negative control (NC), were detected using western blotting. **c** Downregulation of E-cadherin disrupted DNA damage repair pathway and cell cycle checkpoint. Key molecules (ATM, Rad51, ARID1A, yH2AX, CDC2, and CyclinB1) involved in DNA damage and cell cycle checkpoint pathways were examined by western blotting. **d** The effect of E-cadherin knockdown on drug sensitivity (PARP inhibitor and Wee1 inhibitor) in GC cell lines. GC cell lines (N87 and KATO III), infected with si-NC or si-Ecad, were treated with indicated concentration cascades of corresponding drug and vehicle control for 72 h, assessed with CCK-8 assay. The data are expressed as the mean ± SEM of three independent experiments.

As demonstrated by co-occurrence/mutual exclusivity analysis using GO and GSEA methods, the *CDH1*-MT cohort tended to have more gene mutations involved in DDR pathway and cell cycle checkpoint as well as lower expression of genes in G2/M checkpoint and DNA repair pathways. Since most *CDH1* mutations were considered as loss of function, and all the mutations in our study have been confirmed to be pathogenic or likely pathogenic, we knocked down the expression of E-cadherin to explore its biological role in an in vitro model. For this, we selected two GC cell lines with high E-cadherin expression, N87 and KATO III (Fig. 5a, b). Key molecules involved in DDR and cell cycle checkpoints were assessed by western blotting. Our results demonstrated that DDR-related molecules (ARID1A, ATM, Rad51) were significantly inhibited, while DNA damage marker, yH2AX was up-regulated upon E-cadherin knockdown. Meanwhile, cell cycle checkpoints were downregulated, as indicated by the decreased expression of phosphorylated CDC2 merely in both GC cell lines (Fig. 5c), consistent with our GSEA analysis.

Since DDR-defective tumors are more sensitive to PARP inhibitors (i.e., olaparib)^17^ and cell cycle checkpoints are the targets of Wee1 inhibitors (i.e., AZD1775)^18^, we hypothesize that knockdown of E-cadherin expression would sensitize GC cell lines to olaparib or/and AZD1775. Our results demonstrated that the sensitivity to olaparib and AZD1775 was significantly increased by E-cadherin knockdown in both N87 and KATO III GC cell lines (Fig. 5d). Increased sensitivity to cisplatin was also observed in N87 and KATO III after the inhibition of E-cadherin (Supplementary Fig. 3). However, no significant changes of the sensitivity to other chemotherapy drugs (5-Fu and paclitaxel) were seen upon E-cadherin knockdown (Supplementary Fig. 3). These data suggested that inhibition of E-cadherin may sensitize GC cell lines to PARP and Wee1 inhibitors potentially related to disruption of the DDR pathway and cell cycle checkpoints.

We further evaluated the efficacy of the combination of cisplatin, olaparib, and AZD1775 in both E-cadherin-knockdown and control groups of N87 and KATO III. As shown in Supplementary Fig. 4, drug synergies (cisplatin and Olaparib/AZD1775, olaparib and AZD1775) were observed in both E-cadherin-knockdown and control groups of N87 and KATO III.

## Discussion

To the best of our knowledge, our study represents the largest comprehensive report to explore the specific molecular features of *CDH1*-MT GC and identify potential therapy strategies for *CDH1*-MT GC.

The higher frequencies of diffuse histology, the presence of younger patients, and the female predominance observed in the *CDH1*-MT cohort are consistent with the biological functions of *CDH1* in tumorigenesis. Loss of E-cadherin expression is associated with enhanced cellular migration, leading to rapid dissemination of cancer cells in the early stages of tumorogenesis^11^. Cho et al. also reported that somatic *CDH1* mutations were diffuse histology-specific and enriched in early-onset DGC females^19^. The instability of mutant E-cadherin proteins, caused by truncating and missense mutations of *CDH1* (EC1 domain), may lead to the aggregation phenotype^19^. Since normal lymphocyte samples were not available in this retrospective study, we cannot distinguish germline versus somatic *CDH1* mutations. However, the germline mutation rate of *CDH1* in sporadic GC is low. In the TCGA database, no *CDH1* germline mutations were identified in 436 GC patients (http://cbioportal.org). Therefore, our findings characterize**d** the main molecular features of somatic *CDH1*-MT GC. One of our future goals is to compare the different characterizations between somatic and germline mutations of *CDH1* in GC.

Our data demonstrates that positive rates of PD-L1 expression (CPS ≥ 1) are lower in *CDH1*-MT patients than in *CDH1*-WT patients, indicating that *CDH1*-MT GC patients might not benefit from immunotherapy. We reported that *CDH1* mutations are positively associated with mutations of the genes related to chromatin remodeling, including *ARID1A* and *DNMT3A*, and genes related to DNA damage repair, including *CDK12*, *FANCC*, *POT1*, and *WRN*. Consistently, GO enrichment analysis also showed mutated genes involved in DDR pathways were specifically enriched in *CDH1*-mutated GCs. GSEA analysis of TCGA data suggested DDR deficiency and impaired G2/M checkpoint in the *CDH1*-MT cohort, compared to that observed in the *CDH1*-WT cohort. Consistently, EMT, activated by ZEB1 and TGF-β signaling was reported to impair homologous recombination repair (HR) proficiency^20^. bHLH-PAS transcription factor family member single-minded 2 s (SIM2s) is a key regulator of the epithelial phenotype. S115A point mutation was sufficient to induce EMT by inhibiting the expression of E-cadherin. Meanwhile, the S115A mutation can impair the DDR pathway and improve the sensitivity of these tumors to the PARP inhibitor olaparib^21^. In addition, E-cadherin was proven to play a role in efficient DNA repair of UV-induced DNA damage, identifying a new link between epithelial adhesion and DNA repair^22^. Interestingly, *CDH1* germline mutated GCs have higher yH2AX foci, compared to *CDH1*-WT GCs^23^. These phenomena might suggest that E-cadherin loss/*CDH1* mutations could promote tumor initiation through impairing DDR pathways. Additionally, gene mutations involved in the cell cycle checkpoint pathway were also enriched in the *CDH1*-MT cohort compared to the *CDH1*-WT cohort. Our data further confirmed that knockdown of E-cadherin impaired DNA damage repair and cell cycle checkpoint pathways.

HER2 protein expression and amplification were lower in *CDH1*-MT GCs than in *CDH1*-WT GCs, findings which are consistent with the clinical observation that HER2 overexpression and amplification are the main characteristics of intestinal GC, while *CDH1* mutation is the main feature of DGC^7^. These findings suggested clinical trials to develop treatment strategies targeting DDR and cell cycle checkpoint pathways in *CDH1*-MT GC are needed.

It is well-known that PARP inhibitors competitively bind and trap PARP to disrupt single-strand DNA breaks (SSB) repairs^17,24^. Due to the activation of HR induced by the conversion of SSB to double-strand DNA breaks, the efficiency of targeting PARP may be limited in cancer treatment. Therefore, synthetic lethality was proposed as the mechanism of tumor destruction observed by applying PARP inhibitors in patients with tumors that exhibit a deficiency of HR function. Our data showed knockdown of E-cadherin inhibits the key regulators of HR, including RAD51, ATM, and ARID1A. Consequently, loss of E-cadherin was shown to sensitize GC cells to treatment with olaparib, a finding that was also seen in studies of patients with breast cancer. It was reported that olaparib enhances platinum sensitivity. HR-deficient (HRD) tumors also show increased sensitivity to platinum-based chemotherapy^25,26^. These findings could also explain the phenomenon that knockdown of E-cadherin could improve the sensitivity of tumor cells to cisplatin^27^. The phase III GOLD trial failed to show benefit from adding the PARP inhibitor olaparib to paclitaxel in the second line based on ATM expression^28^, suggesting more genomic and functional measures of HRD and replication stress should be explored to predict the response to PARP inhibition. According to our data, *CDH1* mutations may serve as a biomarker to select GC patients who could probably benefit from treatment with olaparib. Platinum sensitivity may itself be a predictive biomarker, a concept that has led to a phase III trial of PARP inhibition versus placebo as maintenance therapy in advanced GC that responded to platinum-based first-line chemotherapy (NCT03427814). The role of *CDH1* mutation in predicting the efficacy of PARP inhibition as a maintenance treatment deserves to be explored in this trial. In addition, the phosphorylation of key molecular CDC2 involved in the G2/M checkpoint was downregulated upon the knockdown of E-cadherin, which can enhance the anti-tumor activity of the Wee1 inhibitor AZD1775 by impairing the G2/M checkpoint and promoting premature mitosis. *TP53* mutation is one of the best-studied predictive biomarkers for Wee1 inhibition in ovarian cancer^18^, but its usefulness remains controversial in GC. Based on our data, *CDH1* mutations may provide insight to better select patients for AZD1775. If these findings could be validated in prospective clinical trials, *CDH1* mutations should be routinely checked to improve the survival of GC patients.

Drug combinations to specifically target cancer-inducing or cell-sustaining pathways are a cornerstone of cancer therapy. Previous studies have revealed that the combination of cisplatin, with Wee1 and PARP inhibitors could significantly augment anti-tumor activity compared to mono-therapy^24,28,29^. Drug synergies (cisplatin and olaparib/AZD1775, olaparib and AZD1775) were observed in both E-cadherin-knockdown and control groups of N87 and KATO III GC cell lines. Therefore, these findings suggest further study of the potential to use the presence of a *CDH1* mutation as a predictive biomarker to select patients who may benefit from maintenance therapy, following the treatment with drug combinations that target tumors that manifest HRD and/or overexpress Wee1 (cisplatin and olaparib/AZD1775, olaparib and AZD1775). Due to the high heterogeneity of GC and our limited data based on two selected GC cell lines, the correlation of *CDH1* mutations to single agent and doublet treatment of AZD1775, cisplatin, and olaparib deserves to be further studied in expanded GC models, such as patient-derived xenografts, which would better compare the efficacy of combined treatment based on the maximum tolerated dose in vivo and help launch drug efficacy trials in mice prior to their introduction in human trials.

Limitations of this work need to be mentioned. The retrospective setting of this study may introduce the selection bias, including the missing information on Lauren’s classification for the majority of samples. Thus, these results need to be validated in prospective clinical trials.

In summary, this is the largest comprehensive molecular characterization of *CDH1*-MT GC patients. We preliminarily identified GC patients with *CDH1* mutations that could potentially benefit treatment targeting DDR and cell cycle checkpoint pathways.

## Methods

### Patients and tumor samples

Data from a total of 1596 patients with pathologically confirmed GC samples and their anonymized clinical records were collected by a commercial CLIA-certified laboratory (Caris Life Sciences, Phoenix, USA) (referred to as CARIS) to examine somatic mutations via targeted sequencing. Among them, tumors from 253 patients underwent whole-transcriptome sequencing to identify genetic fusions. This study was conducted in accordance with the guidelines of the Declaration of Helsinki, the Belmont Report, and the U.S. Common Rule. In keeping with 45 CFR 46.101(b)(4), this study was performed utilizing retrospective, de-identified clinical data. Therefore, this study is considered IRB-exempt and no patient consent was deemed to be necessary.

### Targeted next-generation sequencing

Prior to molecular testing, tumor enrichment was achieved by harvesting targeted tissue from formalin-fixed paraffin-embedded (FFPE) tumor samples using manual microdissection techniques. Matched normal tissue was not sequenced. Next-generation sequencing (NGS) and gene variant calling were performed as previously described^30^. Briefly, genomic DNA was isolated from enriched tumors to generate the capture-based library, followed by Agilent’s SureSelectXT assay (Agilent Technologies, Santa Clara, CA, USA) to enrich 592 whole-gene targets. All variants were detected with >99% confidence based on allele frequency and amplicon coverage, with an average sequencing depth of coverage of >500 and an analytic sensitivity of 5%. Genetic variants identified were interpreted by board-certified molecular geneticists and categorized as ‘pathogenic’, ‘presumed pathogenic’, ‘variant of unknown significance’, ‘presumed benign’, or ‘benign’, according to the American College of Medical Genetics and Genomics (ACMG) standards. When assessing mutation frequencies of individual genes, only ‘pathogenic’, and ‘presumed pathogenic’ were counted as mutations.

A combination of multiple test platforms was used to determine the MSI or MMR status of the tumors profiled, including fragment analysis (FA, Promega, Madison, WI), IHC (MLH1, M1 antibody; MSH2, G2191129 antibody; MSH6, 44 antibody; and PMS2, EPR3947 antibody [Ventana Medical Systems, Inc., Tucson, AZ, USA]) and NGS (for tumors tested with NextSeq platform, 7000 target microsatellite loci were examined and compared to the reference genome hg19 from the University of California). GO analysis of genes (mutation rates ≥ 1%) in the *CDH1*-MT and WT cohorts was conducted using the Database for Annotation, Visualization, and Integrated Discovery Bioinformatics Resources 6.8 (DAVID; http://david.abcc.ncifcrf.gov) respectively. The “TCGA-gastric cancer” dataset was publicly available through the cBioPortal for Cancer Genomics (http://cbioportal.org).

### IHC staining and CISH

IHC was performed on full slides of FFPE tumor specimens as previously described using automated staining techniques^30^. Candidate targets, including PD-L1 (22c3, DAKO, Santa Clara, CA) and HER2 (4B5, Ventana Medical Systems), were stained. IHC staining was categorized as positive or negative based on the intensity and staining percentage, according to the criteria reported previously^30^.

CISH was performed for *HER2* genes using automated staining (Benchmark XT; Ventana, Tucson, AZ) and imaging (BioView, Billerca, MA) techniques. Gene amplifications were defined as the ratio of *HER2*/*CEN17* ≥ 2.0. Both of IHC and CISH results were reviewed and scored by two independent pathologists blinded to each other.

### Gastric cancer cell lines and cell viability assay

The human gastric cancer cell lines AGS, MKN1, MKN74, NUGC3, NUGC4, KATO III, IM95, and N87 were provided by Professor Zev A. Wainberg from the University of California, Los Angeles. All cells were identified by short tandem repeat analysis and confirmed to contain no mycoplasma infection. Cells were cultured in RPMI-1640 (Corning, Manassas, VA) medium containing 10% fetal bovine serum (Gibco BRL) and 1% penicillin and streptomycin (HyClone, Logan, UT), and incubated at 37 °C in a humidified incubator with 5% CO_2_.

Cells (about 4000 cells per well) were plated onto 96-well plates and cultured overnight in a complete medium. Cells were treated with serial concentrations of AZD1775, olaparib, cisplatin, paclitaxel, and 5-Fluorouracil (5-Fu) (MedChem Express, Princeton, NJ, USA) for 72 h, and then evaluated for cell viability using the CCK-8 assay (#CK04, Dojindo, Japan) following the manufacturer’s instructions. The absorbance was measured at 450 nm using a spectrophotometer. All assays were repeated at least three times.

### RNA interference

The expression of E-cadherin was lowered using target-specific small interfering RNA (siRNA) molecules purchased from Horizon Discovery (Cambridge, UK) as follows: Control siRNA (D-001810-10-05), E-cadherin siRNA (LQ-003877-08, LQ-003677-09). Gene-specific or control siRNA was transfected into cells at 60–70% confluence using Lipofectamine™ 3000 reagent (Thermo Fisher, USA) according to the manufacturer’s instructions.

### Western-blotting analysis

The extraction of total protein from cell pellets and western blotting was conducted as previously described^31^. Proteins were visualized using Azure Digital Imaging System C300 (Azure Biosystems, Dublin, CA, USA). Antibodies for E-cadherin (#3195), GAPDH (#5174), ARID1A (#12354), ATM (#2873), Rad51 (#8875), yH2AX (#7631), pCDC2 (#4539) and Cyclin B1 (#D5C10) were purchased from Cell Signaling technology. Uncropped scans were supplied in Supplementary Fig. 5.

### Statistical analysis

Statistical analysis was performed with R version 4.3.1, Excel, or GraphPad 7.0. The comparisons of continuous data were analyzed using the Student’s t-test or the Kruskal-Wallis test, and those of categorical data were analyzed using the Chi-square test or the Fisher test. OS was defined as the time from sample collocation until death or the end of follow-up. IrOS was defined as the time from initial immunotherapy (nivolumab or pembrolizumab treatment) to the day of death or the end of follow-up. Ramucirumab-related OS was defined as the time from initial ramucirumab to the day of death or the end of follow-up. GSEA was performed with GSEA software (v4.0.1). GO and KEGG pathway enrichment for transcriptome analysis were conducted online (g:Profiler; www.biit.cs.ut.ee/gprofiler/gost). A two-sided *p* < *0.05* was considered statistically significant.

## Supplementary information

Supplementary table and figures

## Data Availability

The datasets generated and/or analyzed during the current study are available for replication and verification purposes from the corresponding author upon reasonable request. The de-identified DNA sequencing data are owned by Caris Life Sciences and cannot be publicly shared due to the data usage agreement signed by Dr. Heinz-Josef Lenz at Keck School of Medicine of USC. Qualified researchers can apply for access to these data by contacting Joanne Xiu, PhD at jxiu@carisls.com, submitting a brief proposal, and signing a data usage agreement. DNA and RNA-sequencing data from the TCGA cohort were available on the website (www.cbioportal.org).
