# Supplementary material for: Large-scale analysis of CDH1 mutations defines a distinctive molecular subset with treatment implications in gastric cancer
Source: NPJ Precis Oncol. 2024 Sep 30;8:214. doi: 10.1038/s41698-024-00694-8 (PMC11442451; doi:10.1038/s41698-024-00694-8)
Supplement: Supplementary file 1 — Supplementary table and figures [file 41698_2024_694_MOESM1_ESM.pdf]

Supplementary Figure 1

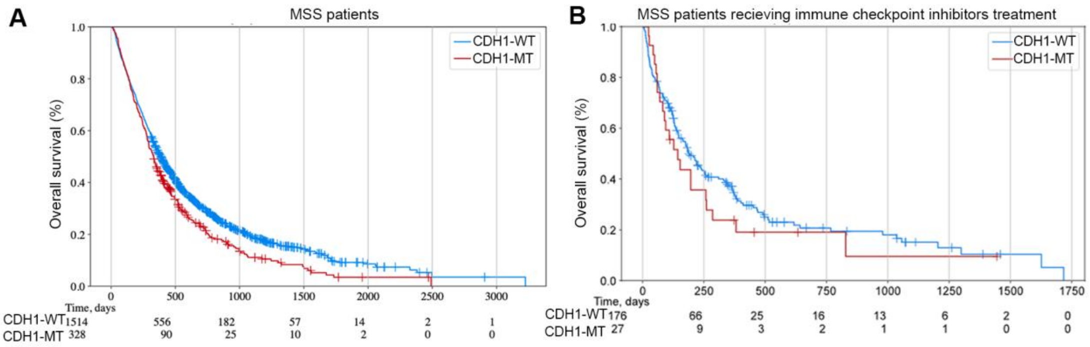

Supplementary Figure 1. The association of *CDH1* mutations with overall survival (A) and immune checkpoint inhibitor-related survival (B) in patients with microsatellite stable (MSS) gastric cancers.

Supplementary Figure 2

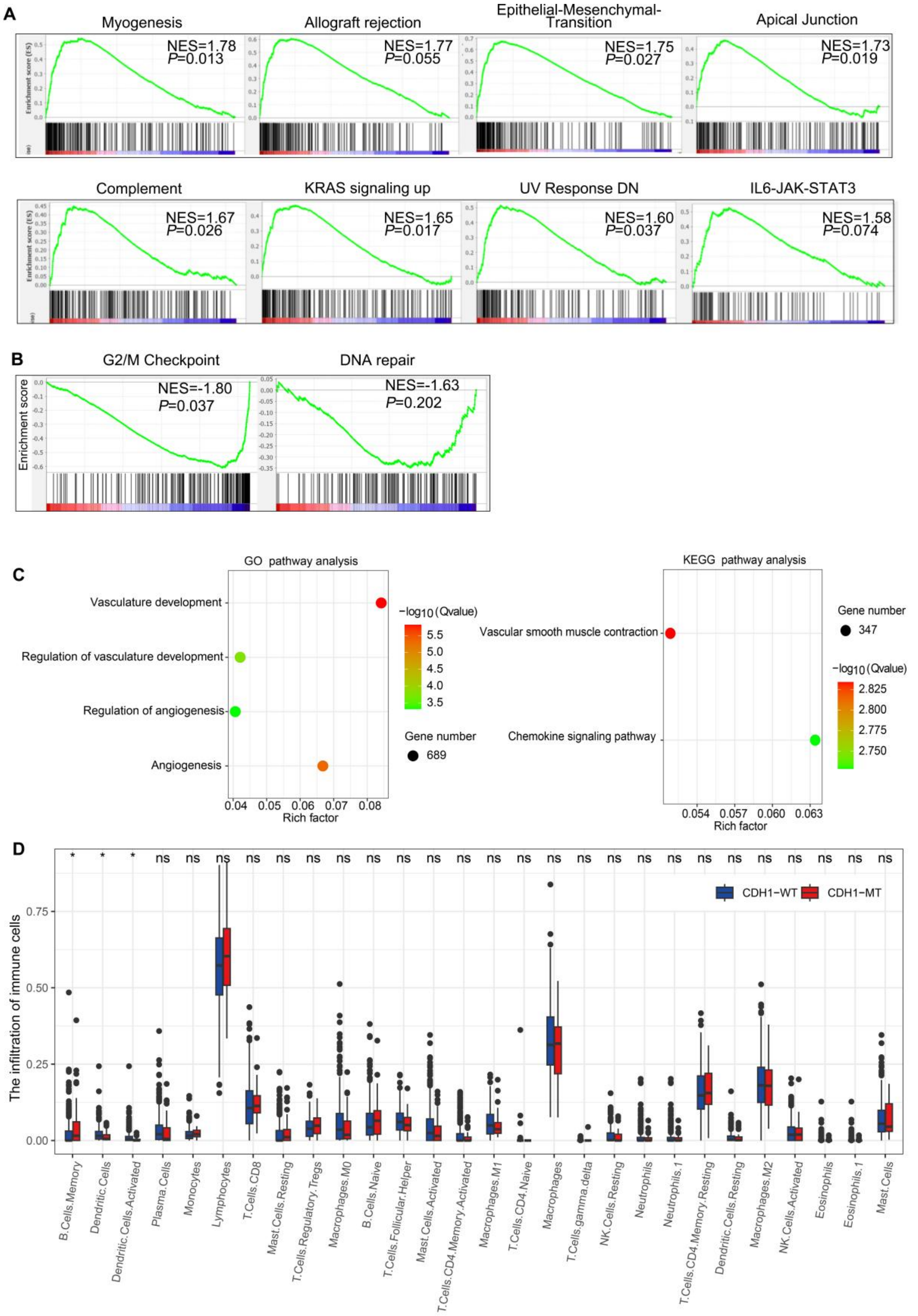

Supplementary Figure 2. A. GSEA enrichment analysis of hallmark reference gene sets for TCGA cohort according to *CDH1* mutation status. Top 8 enriched pathways were shown. **B.** G2/M checkpoint and DNA repair pathways were

enriched in *CDH1*-WT GC, compared to *CDH1*-MT GC. **C.** Angiogenesis-related pathways were enriched in *CDH1*-mutant GC patients using GO and KEGG analysis, based on the TCGA cohort. **D.** Impact of *CDH1* mutations on the infiltration of immune cells (the proportions of immune cells within the leukocyte compartment) in patients with gastric cancer in the TCGA cohort, estimated using CIBERSORT. NES, normalized enrichment score. GO, Gene Ontology. KEGG, Kyoto Encyclopedia of Genes and Genomes.

Supplementary Figure 3

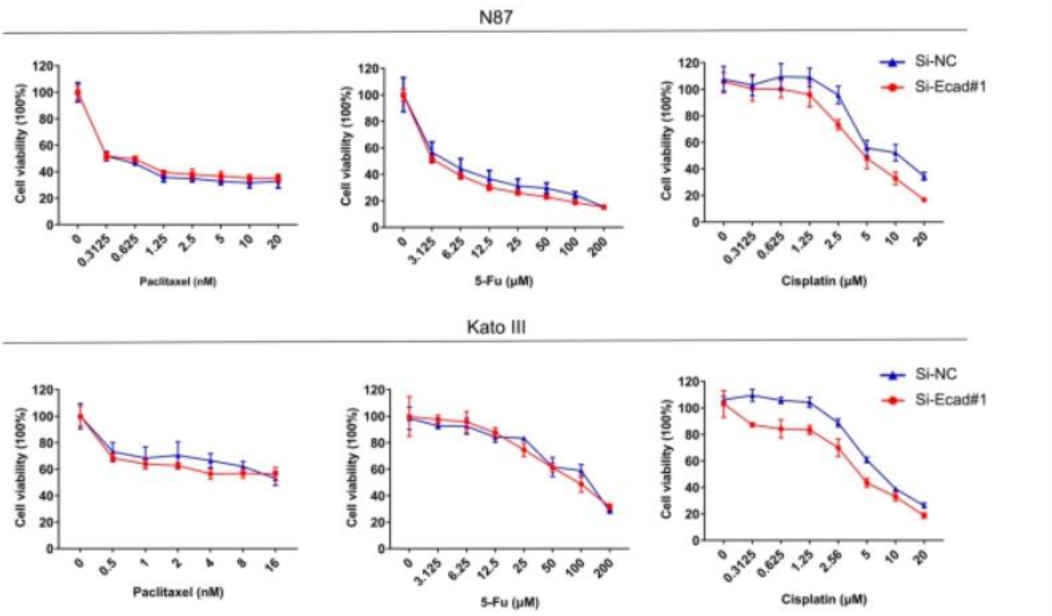

Supplementary Figure 3. The effect of E-cadherin knockdown on drug sensitivity to chemotherapy (paclitaxel, 5-Fu, and cisplatin) in GC cell lines. GC cell lines (N87 and KATO III), infected with si-NC or si-Ecad, were treated with indicated concentration cascades of corresponding drug and vehicle control for 72h, assessed with CCK-8 assay. The data are expressed as the Mean ± SEM of three independent experiments.

Supplementary Figure 4

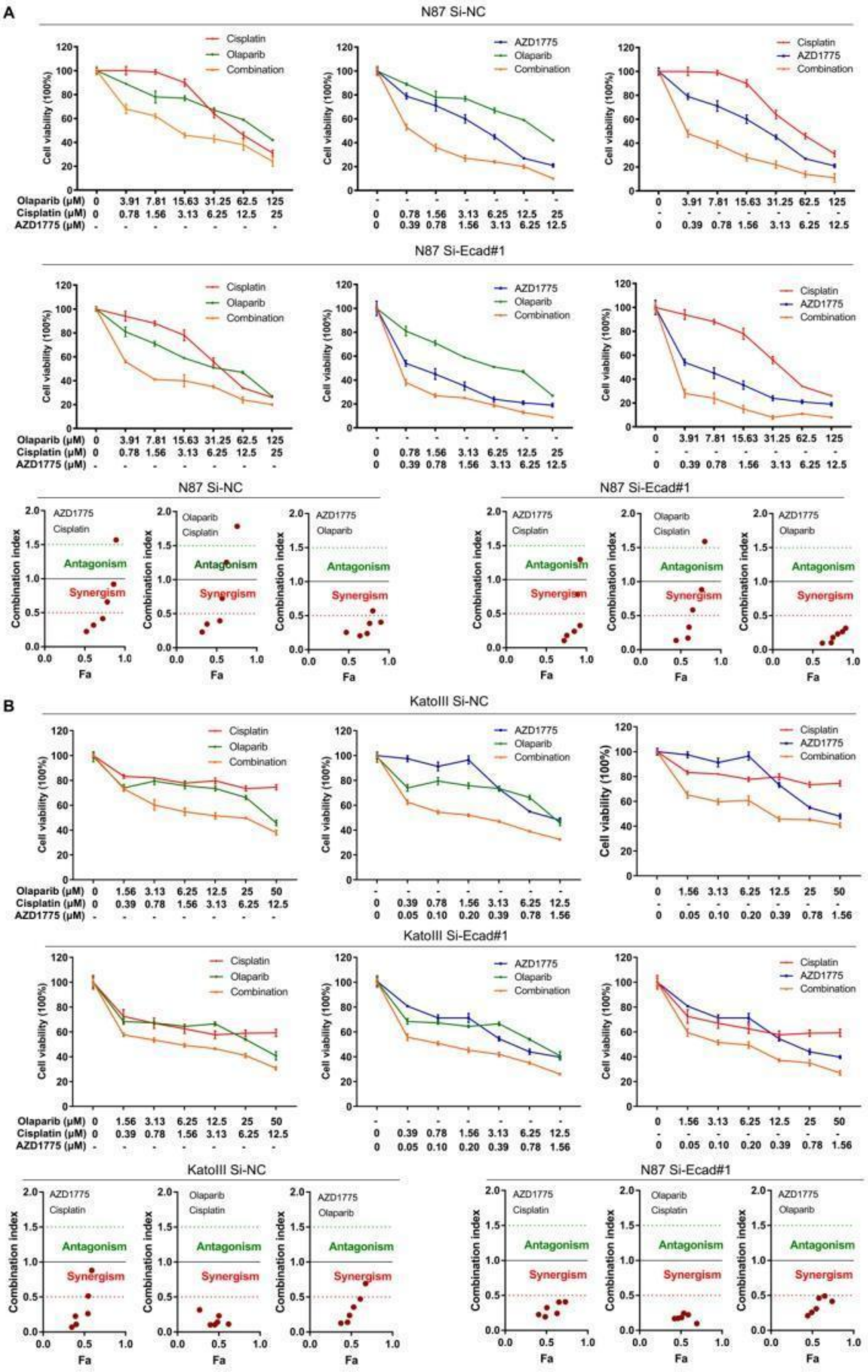

Supplementary Figure 4. The efficacy of drug combinations (AZD1775, olaparib and cisplatin) in N87 (A) and KATO III (B). GC cell lines (N87 and KATO III), infected with si-NC or si-Ecadherin (Ecad), were treated with

indicated concentration cascades of corresponding monotherapy of (cisplatin, olaparib and AZD1775) and combined therapy (cisplatin and olaparib, olaparib and AZD1775, cisplatin and AZD1775) for 72h, assessed with CCK-8 assay. The data are expressed as the Mean ± SEM of three independent experiments.

Supplementary Figure 5.

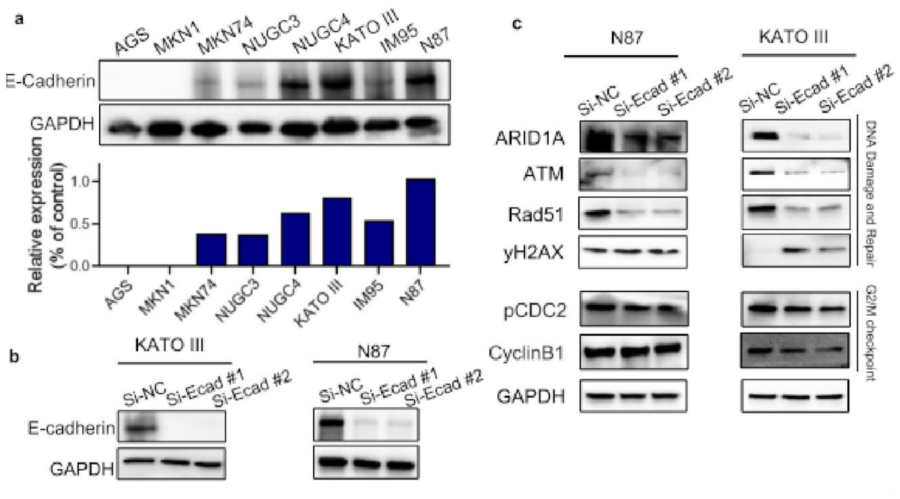

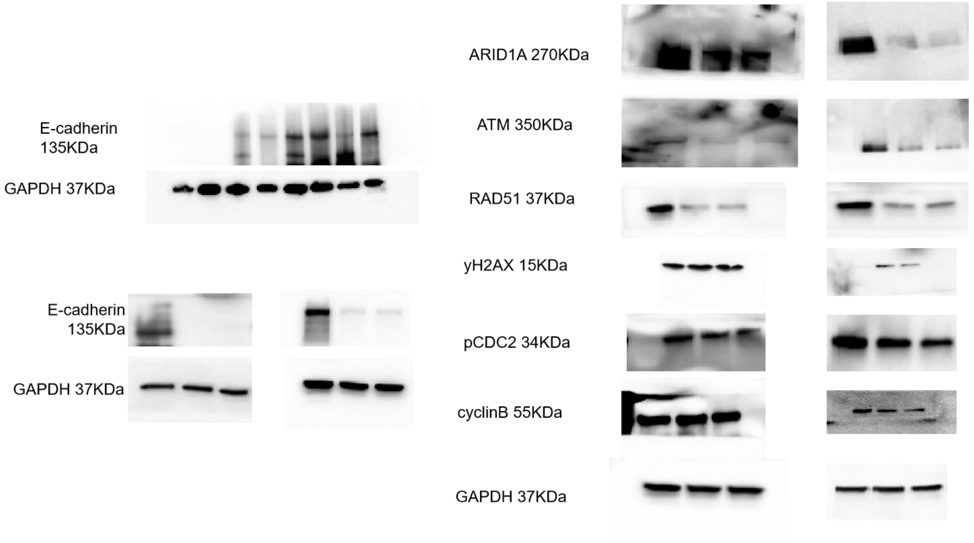

Supplementary Figure 5. Uncropped scans were provided.

Supplementary **Table 1. Baseline demographic characteristics.**

Clinicopathological feature		CARIS (N=1,596)	TCGA (N=436)	*P*
		N (%)	N (%)	
Age[1]	Median	63	67	<0.001
	Range	(12~94)	(30~90)	
Gender	Male	1,021 (64.0%)	280 (64.2%)	0.924
	Female	575 (36.0%)	156 (35.8%)	
Lauren subtype	Diffuse	123 (7.7%)	85 (19.5%)	<0.001
	Intestinal	73 (4.6%)	190 (43.6%)	
	Mixed or unclear	1,400 (87.7%)	161 (36.9%)	
Tumor site	Primary	987 (61.8%)	436 (100%)	<0.001
	Metastasis	584 (36.6%)	0 (0%)	
	NA[2]	25 (1.6%)	0 (0%)	
MSI status	MSI	100 (6.3%)	73 (16.7%)	<0.001
	MSS	1,489 (93.3%)	310 (71.1%)	
	NA	7 (0.4%)	53 (12.2%)	

[1]Median (range);

[2] NA: Not available.

*A two-sided $P < 0.05$ was considered statistically significant. *P* was calculated by chi-square test, unpaired two-tailed t-test or one-way analysis of variance separately.

Supplementary Table 2. The association between *ARHGAP26*-fusion and *CDH1* mutation.

	ARHGAP26-fusion n (%)	*ARHGAP26*-wild-type n (%)	*P*
CDH1 mutation	2 (7.69)	24 (92.31)	0.906
CDH1 wildtype	19 (8.37)	208 (91.63)	

Supplementary Table 3. Functional terms by GO enrichment in *CDH1*-MT and WT cohort.

Cohort	Term	FDR
CDH1-MT	Chromosome organization	3.24E-09
CDH1-MT	Regulation of apoptosis	1.18E-07
CDH1-MT	Regulation of programmed cell death	1.39E-07
CDH1-MT	Response to DNA damage stimulus	1.70E-07
CDH1-MT	DNA recombination	3.93E-07
CDH1-MT	Regulation of cell proliferation	7.37E-07
***CDH1*-MT**	**DNA metabolic process**	**8.79E-07**
***CDH1*-MT**	**DNA repair**	**1.18E-06**
CDH1-MT	Cellular response to stress	4.10E-06
CDH1-MT	Regulation of cell cycle	6.68E-06
CDH1-MT	Regulation of mitotic cell cycle	1.07E-05
CDH1-MT	Immune system development	1.22E-05
CDH1-MT	Regulation of DNA metabolic process	2.09E-05
CDH1-MT	Induction of apoptosis by intracellular signals	9.87E-05
CDH1-MT	Anatomical structure homeostasis	2.78E-04
CDH1-MT	Cell cycle process	3.03E-04
CDH1-MT	Intracellular signaling cascade	0.00105
CDH1-MT	DNA damage response, signal transduction	0.00106
CDH1-MT	Response to radiation	0.00162
CDH1-MT	Cell cycle	0.0018
CDH1-MT	Negative regulation of cell death	0.00181
***CDH1*-MT**	**Cell cycle checkpoint**	**0.00228**
CDH1-MT	Response to drug	0.0029
CDH1-MT	Phosphoinositide 3-kinase cascade	0.00321
CDH1-MT	Protein complex biogenesis	0.00528
CDH1-MT	Protein complex assembly	0.00528
CDH1-MT	Somatic diversification of immune receptors	0.00563
CDH1-MT	Cell cycle phase	0.00634
CDH1-MT	Telomere maintenance	0.00655
***CDH1*-MT**	**Double-strand break repair**	**0.00679**
CDH1-MT	Aging	0.00692
CDH1-MT	Telomere organization	0.00757
CDH1-MT	Immunoglobulin production	0.00871
CDH1-MT	Production of molecular mediator of immune response	0.00997
CDH1-MT	Chromatin modification	0.0168
CDH1-MT	Response to hormone stimulus	0.01818
CDH1-MT	Response to abiotic stimulus	0.01858
CDH1-MT	Positive regulation of molecular function	0.02173
CDH1-MT	Macromolecular complex subunit organization	0.02375

CDH1-MT	Regulation of cell adhesion	0.0245
CDH1-MT	Mitotic cell cycle checkpoint	0.03777
CDH1-MT	Response to endogenous stimulus	0.03978
CDH1-MT	Negative regulation of nucleobase, nucleoside, nucleotide and nucleic acid metabolic process	0.04058
CDH1-MT	Positive regulation of catalytic activity	0.04634
CDH1-WT	Regulation of cell proliferation	2.68E-10
CDH1-WT	Chromosome organization	8.95E-09
CDH1-WT	Cell cycle process	9.09E-09
CDH1-WT	Cell cycle	1.95E-07
CDH1-WT	Regulation of mitotic cell cycle	8.95E-07
CDH1-WT	Regulation of cell cycle	1.07E-06
CDH1-WT	Cell cycle phase	1.50E-06
CDH1-WT	Regulation of apoptosis	2.19E-05
CDH1-WT	Regulation of programmed cell death	2.53E-05
CDH1-WT	Negative regulation of cell death	3.36E-05
CDH1-WT	Response to hormone stimulus	4.16E-05
CDH1-WT	Response to endogenous stimulus	1.23E-04
CDH1-WT	Response to radiation	1.92E-04
CDH1-WT	Immune system development	2.72E-04
CDH1-WT	Response to drug	3.71E-04
***CDH1*-WT**	**Transmembrane receptor protein tyrosine kinase signaling pathway**	**5.05E-04**
CDH1-WT	Intracellular signaling cascade	5.25E-04
CDH1-WT	Regulation of epithelial cell proliferation	7.31E-04
CDH1-WT	Response to steroid hormone stimulus	0.00185
CDH1-WT	Enzyme linked receptor protein signaling pathway	0.00195
CDH1-WT	Regulation of cell adhesion	0.00236
CDH1-WT	Chromatin modification	0.00272
CDH1-WT	Response to abiotic stimulus	0.00377
CDH1-WT	Phosphoinositide 3-kinase cascade	0.00382
CDH1-WT	Negative regulation of cell differentiation	0.00448
***CDH1*-WT**	**Regulation of cyclin-dependent protein kinase activity**	**0.00451**
CDH1-WT	Regulation of transcription	0.00555
CDH1-WT	Positive regulation of molecular function	0.00623
CDH1-WT	Regulation of cell-matrix adhesion	0.00707
***CDH1*-WT**	**Response to estrogen stimulus**	**0.00738**
CDH1-WT	DNA recombination	0.00738
CDH1-WT	Anatomical structure homeostasis	0.00779
CDH1-WT	Positive regulation of catalytic activity	0.01239
CDH1-WT	Regulation of protein kinase activity	0.01778
CDH1-WT	Protein amino acid phosphorylation	0.02324
***CDH1*-WT**	**Regulation of kinase activity**	**0.02337**
CDH1-WT	Lymphocyte activation	0.02777

CDH1-WT	Mitotic cell cycle	0.03105
CDH1-WT	DNA damage response, signal transduction	0.03159
CDH1-WT	Regulation of transferase activity	0.03241
CDH1-WT	Response to DNA damage stimulus	0.0331
CDH1-WT	Response to light stimulus	0.03546
CDH1-WT	Cell activation	0.0359
CDH1-WT	Positive regulation of transcription, DNA-dependent	0.03681
CDH1-WT	Positive regulation of rna metabolic process	0.03955
CDH1-WT	Regulation of phosphorus metabolic process	0.04246
CDH1-WT	Mitotic cell cycle checkpoint	0.04735
CDH1-WT	Phosphate metabolic process	0.04959
CDH1-WT	Phosphorus metabolic process	0.04959

*FDR < 0.05 was considered statistically significant. Bold lines were selected to be shown in Figure 2C.
